# Fermentative Succinate Production: An Emerging Technology to Replace the Traditional Petrochemical Processes

**DOI:** 10.1155/2013/723412

**Published:** 2013-12-12

**Authors:** Yujin Cao, Rubing Zhang, Chao Sun, Tao Cheng, Yuhua Liu, Mo Xian

**Affiliations:** CAS Key Laboratory of Bio-Based Materials, Qingdao Institute of Bioenergy and Bioprocess Technology, Chinese Academy of Sciences, Qingdao 266101, China

## Abstract

Succinate is a valuable platform chemical for multiple applications. Confronted with the exhaustion of fossil energy resources, fermentative succinate production from renewable biomass to replace the traditional petrochemical process is receiving an increasing amount of attention. During the past few years, the succinate-producing process using microbial fermentation has been made commercially available by the joint efforts of researchers in different fields. In this review, recent attempts and experiences devoted to reduce the production cost of biobased succinate are summarized, including strain improvement, fermentation engineering, and downstream processing. The key limitations and challenges faced in current microbial production systems are also proposed.

## 1. Introduction

Succinate, also known as 1,4-butanedioic acid or amber acid, is a four-carbon dicarboxylic acid for multiple applications. Succinate and its desirable properties have been known for a long time. In the agricultural field, succinate is a known growth regulator [1] which can be used for seed treatment and plant rooting. In the food industry, succinate is used as a flavoring enhancer for beverages, a bread softening agent, and a catalyst for food seasoning preparation [2]. It is generally considered as safe and has been approved by the US Food and Drug Administration. In the pharmaceutical industry, succinate acts as an anticarcinogenic agent and as an insulinotropic agent [3]. In the chemical industry, succinate is a precursor for the production of many high-value chemicals including 1,4-butanediol, tetrahydrofuran, *γ*-butyrolactone, and 2-pyrrolidinone [4]. Finally, succinate is a precursor to many specialized polyesters, for example, polybutylene succinate (PBS) [5], which might be its most promising application area.

**Figure 1:**
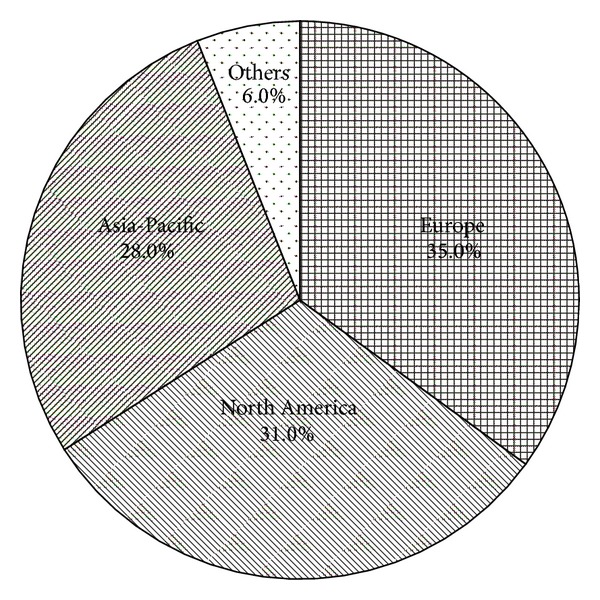
Succinate market share by geography in the year of 2010.

Due to its versatile applications, succinate is rising to a bulk chemical in recent years. The global production is estimated between 30,000 and 50,000 tons per year [6]. According to a survey report from MarketsandMarkets, the market of succinate is expected to grow at a rate of 18.7% from 2011 to 2016 [7]. The global market for succinate in terms of revenue was estimated to be worth $182.8 million in 2010 and is expected to reach $496.0 million by 2016. As shown in Figure 1, Europe and North America were the largest two markets in 2010, accounting for 35.0% and 31.0% of the global succinate demand, respectively. Asia-Pacific was the third succinate consuming regions and is expected to be the fastest growing market in the near future owing to the strong demand from key countries such as China and India. The increasing demand of succinate is promoting us to develop cost effective synthesis routes to support its ever-growing markets.

## 2. The Limitation of Traditional Petrochemical Succinate-Producing Processes

Succinate is traditionally manufactured through chemical routes using paraffin, maleic anhydride, acetylene, or acrylic acid as the starting materials. Paraffin oxidation is the initial method to prepare succinate [8]. Under the catalysis of Mn or Ca, paraffin is deeply oxidized to a mixture of dicarboxylic acid. After steam distillation, succinate in the aqueous phase can be crystallized. This process is well established, but the yield and purity of succinate are relatively low. Another conventional approach widely used to produce succinate is the hydrogenation of maleic anhydride. This process requires several types of noble metal-based catalysts, such as Pd and Ru [9]. In addition, the reaction is carried out under high temperature and high pressure conditions [10], which is not environmental friendly. Electrolytic reduction of maleic anhydride in acidic medium also leads to the production of succinate. The reaction could be conducted under mild conditions and the conversion rate is even higher [11]. However, the consumption of large quantities of electricity power increases the production cost.

Although other chemical synthesis routes such as the catalytic addition of acetylene and acrylic acid using much cheaper feedstocks have been proposed [12], their industrial application is still far from occurring. Along with the decrease of global fossil fuel resources and the rise of crude oil price, these traditionally succinate-producing methods from petrochemicals are becoming costly and causing serious pollution problems, requiring us to find new pathways for succinate manufacture.

## 3. Metabolic Pathways for Succinate Production by Microbial Fermentation

In the biological systems, succinate is an essential intermediate for cellular metabolism. In addition, it can also be one of the end-products of anaerobic fermentation. Therefore, it is possible to produce succinate using biological processes to substitute the petrochemicals processes. Biological processes are particularly attractive since microorganisms usually utilize renewable feedstocks and produce much fewer toxic by-products. In a report from the US Department of Energy, succinate is listed as the first among the top 12 value-added chemicals manufactured from biomass [13]. Recently, biological routes for succinate production developed by two companies, BioAmber and Reverdia, are commercially available. These processes are based on proprietary *Escherichia coli* and yeast strains.

**Figure 2:**
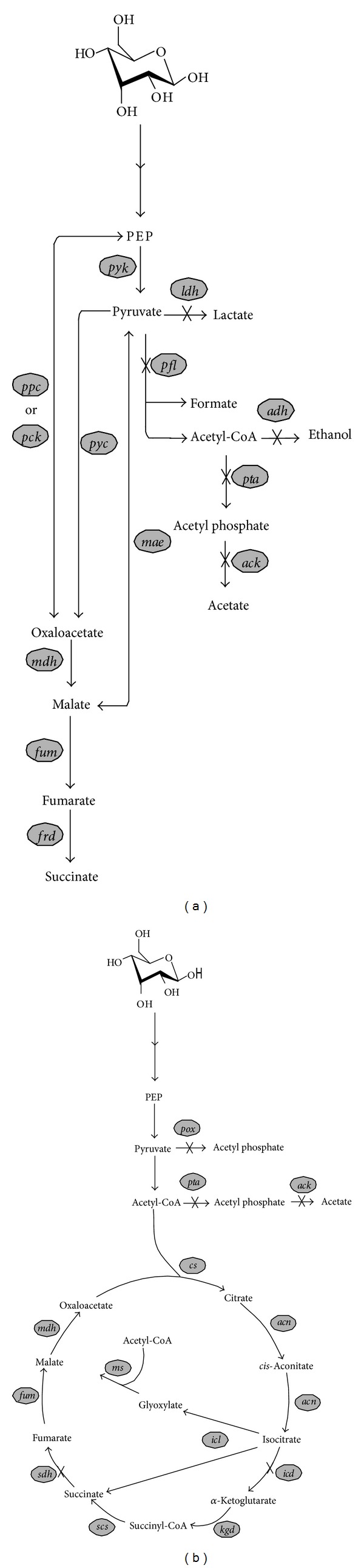
Anaerobic (a) and aerobic (b) metabolic pathways for the biosynthesis of succinate. Unidirectional arrows indicate that the reactions are irreversible while two-directional arrows indicate that the reactions are reversible. Enzymes whose genes are deleted are indicated by “X” across arrows. The abbreviations for the enzymes catalyzing these reactions are *ack*, acetate kinase; *acn*, aconitase; *adh*, alcohol/acetaldehyde dehydrogenase; *cs*, citrate synthase; *frd*, fumarate reductase; *fum*, fumarase; *icd*, isocitrate dehydrogenase; *icl*, isocitrate lyase; *kgd*, *α*-ketoglutarate dehydrogenase; *ldh*, lactate dehydrogenase; *mae*, malic enzyme; *mdh*, malate dehydrogenase; *ms*, malate synthase; *pck*, PEP carboxykinase; *pfl*, pyruvate-formate lyase; *pox*, pyruvate oxidase; *ppc*, PEP carboxylase; *pta*, phosphotransacetylase; *pyc*, pyruvate carboxylase; *pyk*, pyruvate kinase; *scs*, succinyl-CoA synthetase; and *sdh*, succinate dehydrogenase.

Figure 2 provides an overview of the biosynthetic pathway of succinate from glucose under both anaerobic and aerobic conditions. Under both conditions, glucose is degraded into phosphoenolpyruvate (PEP) and finally to pyruvate by glycolysis. Through anaerobic fermentation, PEP is the substrate for a carboxylase-catalyzed anaplerotic reaction and can be converted to oxaloacetate by PEP carboxylase (*ppc*) or PEP carboxykinase (*pck*). Pyruvate, the end-product of glycolysis, can also be incorporated with CO_2_ by pyruvate carboxylase (*pyc*) or malic enzyme (*mae*), forming oxaloacetate or malate. Both oxaloacetate and malate may be further converted to fumarate by malate dehydrogenase (*mdh*) and fumarase (*fum*). Fumarate can be finally reduced to succinate by fumarate reductase (*frd*) [14].

Under aerobic conditions, succinate is an intermediate of both tricarboxylic acid (TCA) cycle and glyoxylate shunt, but no wild-type microorganism could accumulate it in large quantities. Recently, the oxidative pathway for succinate production has been artificially constructed in *E. coli* [15], *Saccharomyces cerevisiae* [16], and *Corynebacterium glutamicum* [17] by deleting the critical gene, succinate dehydrogenase (*sdh*), in the TCA cycle. As two carbons are lost as CO_2_ in the TCA cycle, the glyoxylate shunt is employed to bypass the steps in the TCA cycle to improve the atom economy. As shown in Figure 2(b), the TCA cycle and the glyoxylate shunt separate at the isocitrate point. In order to block the metabolic flux through the TCA cycle, two key regulatory points, *sdh* and isocitrate dehydrogenase (*icd*), must be disrupted. Thus, isocitrate enters the glyoxylate shunt and undergoes cleavage into succinate and glyoxylate, which is catalyzed by isocitrate lyase (*icl*) [18]. Then glyoxylate condenses with acetyl-CoA, yielding malate. Finally, malate enters the residual TCA cycle to regenerate isocitrate. For both anaerobic and aerobic metabolism, branch pathways leading to the formation of formate, acetate, ethanol, and lactate also exist to compete with the succinate-producing pathway.

## 4. Microbial Succinate Producers

Since the succinate metabolic pathway has been resolved in different biological systems, it is possible to produce this valuable chemical through microbial fermentation. Up to now, a number of fermentative succinate-producing bacteria have been isolated and characterized to some extent. These microorganisms can be generally classified into two categories: natural producers and engineered producers. Several representative species that have been extensively investigated are listed in Table 1.

### 4.1. Natural Producers

In nature, many wild-type microorganisms are able to produce succinate at high yields. They are mainly belonging to rumen bacteria including facultative anaerobe (*Actinobacillus succinogenes* [19] and* Mannheimia succiniciproducens* [20]) and strict anaerobe (*Anaerobiospirillum succiniciproducens* [21]). These natural producers show excellent tolerance to osmotic pressure caused by high level of succinate. However, the cultivation of these natural producers always requires expensive nutrient media, thus increasing the production cost.

Many fungal strains such as *Paecilomyces varioti* [22], *Aspergillus niger* [23], and *Penicillium simplicissimum* [24] are also found to be suitable for succinate production. They could secrete succinate as a metabolic by-product under aerobic and/or anaerobic conditions, but the productivity is much lower when compared with the bacterial strains. Besides, succinate is synthesized in the fungal cell mitochondria and has to cross the mitochondrial and cellular membrane [25]. Therefore, it is more favorable to use bacteria for succinate production instead of fungi.

### 4.2. Engineered Producers

In addition to the natural producers, many microorganisms can be metabolically engineered to produce succinate as a fermentative end-product. These engineered producers are always model microorganisms since they are easy to be genetically modified. A completely engineered pathway is required to render them capable of producing succinate. *E. coli*, *C. glutamicum*, and *S. cerevisiae* are the representatives of these engineered producers. As a reference organism, *E. coli* is favored by molecular biologists due to its fast growth rate and ease of manipulation [26, 27]. Numerous studies have been conducted on this bacteria and commercial production of succinate has been reached recently. *C. glutamicum* is one of the few gram-positive bacteria which have been tested for succinate production. Perhaps the highest titer of succinate was obtained in a cell recycling fed-batch culture of this bacterium [28]. *S. cerevisiae* has been well characterized to achieve high concentrations of succinate to enhance the quality of wine [29]. It is also a potential industrial producer since this yeast could grow under acidic conditions.

## 5. Strain Improvement to Increase Succinate Productivity

A highly productive strain is the primary factor to achieve an industrial level succinate production. Early studies for strain improvement mainly relied on identifying and isolating new microbial strains as well as enhancing the succinate-producing ability of currently available strains. Many assay methods for rapid and high-throughput screening of succinate-producing strains were established [30]. However, strain screening always requires considerable amount of time and resources. Along with the development of modern biology technology, the molecular mechanisms for biological succinate production have been largely resolved. It is now possible to rationally modify the microbial strains to improve succinate productivity by metabolic engineering tools. Eliminating the competing pathways, altering metabolic flux to channel the flow of key intermediates, and enhancing cofactors supplies would lead to even higher succinate yields [31]. Many studies have been performed on these topics and encouraging results have been obtained during the past few years. In Table 2, we summarize these advances to improve succinate production using different microorganisms.

### 5.1. Inactivation of the Branch Pathways

As succinate is not the sole product of microbial fermentation, knockout of the enzymes in competitive pathways is an obvious strategy to boost its production. During mixed-acid fermentation under anaerobic conditions, acetate, ethanol, lactate, formate, pyruvate, and succinate make up the major soluble products [51]. The first approach to eliminate these by-products was inactivating the lactate producing pathway since this pathway was simply catalyzed by a single enzyme, lactate dehydrogenase (*ldh*) [32]. By deleting the genes encoding the known pathway of acetate formation, phosphotransacetylase (*pta*), and acetate kinase (*ackA*), the synthesis of this by-product could be drastically reduced and succinate formation was enhanced [49]. The branch pathway splitting pyruvate into acetyl-CoA and formate by means of pyruvate formate lyase (*pfl*) would lead to the formation of acetate, ethanol, and formate. However, knockout of the *pfl* gene individually could only enhance lactate production while the succinate concentration in the *pfl* mutant strain was even slightly lower when compared to the control strain [52]. Although the *ldh* and *pfl* double mutant strain could lead to an improved succinate production to about 28.2 g/L, this strain lost its ability to ferment glucose anaerobically due to redox imbalance [33]. The introduction of another null mutation of the *ptsG* gene which encoded a key enzyme for the PEP-dependent phosphotransferase system (PTS) essential to sugar uptake into the double mutant strain restored the ability to ferment glucose and the resulting strains produced more succinate [34]. Fed-batch culture using the triple mutant strain as the host achieved a final succinate concentration of 99.2 g/l with an overall yield of 110% and productivity of 1.3 g/(L *·* h) [53]. In addition, pyruvate formation could be inhibited by disrupting two pyruvate kinases (*pykF* and *pykA*) together with the *ptsG* gene, which also allowed enhanced succinate production [35].

For aerobic succinate production, the TCA cycle can be blocked at key points for the accumulation of succinate. The disruption of the sdh and icd genes is indispensable to create an aerobic succinate-producing strain [36]. The glyoxylate route, which is critical for aerobic succinate production, could be activated by knocking out a repressor protein, IclR [37]. Besides, pyruvate oxidase (*pox*), catalyzing the oxidation of pyruvate, leads to the production of acetate under aerobic conditions. Knockout of this gene was also shown to be helpful for the production of succinate and succinate yield reached 1.2 moles per moles of glucose [38].

### 5.2. Redirecting Metabolic Flux to the Intermediates of Succinate-Producing Pathway

Overexpression of the genes directly involved in the succinate biosynthesis pathway also shows the potential to enhance its production. The genes coding for key enzymes responsible for succinate biosynthesis have been identified to be *ppc*, *pck*, *pyc*, *mae*, *mdh*, *fum*, and *frd*. There are three different kinds of CO_2_-fixing metabolic reactions catalyzed by *ppc*, *pck*, and *pyc*, respectively. These carboxylation reactions of three-carbon metabolites are the first step for succinate synthesis. Expression of these three key enzymes has been demonstrated to be critical for the enhancement of succinate production in different hosts [28, 39, 40, 50]. Mae, catalyzing the reductive carboxylation of pyruvate to malate, provides an alternative route to succinate from pyruvate instead of PEP. When expressing an NAD^+^-dependent malic enzyme in a mutant *E. coli* strain that was unable to metabolize pyruvate, fermentative metabolism was redirected to succinate and thus resulting in accumulation of succinate [41]. Mdh catalyzes the conversion of oxaloacetate to malate. Overexpressed *mdh* in the *ldh* and *pfl* double mutant strain could restore anaerobic cell growth and glucose utilization. About 21 g/L of glucose was completely consumed and succinate reached 15.2 g/L after anaerobic fermentation for 15 h [42]. Fum could convert malate to fumarate and further overexpression of this enzyme with *mae* eliminated malate production while enhancing succinate production [43]. Frd catalyzes the reduction of fumarate to succinate, the last step for succinate production. It plays a key role in the energy metabolism and also contributes to succinate production during microbial fermentation [54].

### 5.3. Enhancement of Reducing Power Availability and Energy Metabolism

When manipulating metabolic fluxes to specific metabolites, it is important to achieve a redox and energy balance between the substrates and the products. Succinate biosynthesis is strongly determined by the reducing power and energy supplies of the host. The anaerobic fermentative pathway from PEP or pyruvate to succinate requires two moles of NADH [55]. The reducing power generated in the formation of PEP or pyruvate through the glycolytic pathway (one NADH) is not enough to convert all the PEP to succinate. Therefore, supplying additional reducing power has the potential to enhance succinate production [56]. A higher NADH/NAD^+^ ratio could be obtained through manipulating the culture to a more reductive environment [57]. By eliminating the IclR transcriptional repressor to activate the aerobic glyoxylate pathway in a mutant strain, enough NADH is generated to reduce the metabolic intermediates to succinate. This engineered strain was capable of achieving a succinate yield of 1.6 moles per mole of glucose at very high rates [44]. On the contrary, excessive supply of NADH might also inhibit succinate production. It is crucially important to maintain a redox balance in the host. For instance, NAD^+^ regeneration was achieved in the *pfl* and *ldh* double mutant strain by overexpression of nicotinate phosphoribosyltransferase. A significant increase in cell mass and succinate production were observed. The final titer was enhanced to 3.7-fold of the control strain under the same induction conditions [45].

ATP supply is another critical factor that influences the production of succinate. The conversion of PEP to oxaloacetate can be catalyzed by *ppc* and *pck*, respectively. Energy contained in PEP is lost using the former carboxylase with the release of inorganic phosphate. On the contrary, the reaction catalyzed by the ATP-generating *pck*, which was found in *A. succiniciproducens* [58], is favorable for succinate biosynthesis. Heterologous expressing this carboxykinase in an *E. coli ppc* mutant strain produced additional ATP and increased succinate production by 6.5-fold [46].

## 6. Fermentation Process Engineering

Fermentation engineering is the foundation for the industrialization of biobased succinate. The succinate fermentation process has been investigated in depth in the past few decades and recent work mainly focused on the following issues.

### 6.1. Optimization of Fermentation Conditions

Media components and fermentation process parameters are the most basic and simplest approach to achieve a higher titer of the desired products. Since the media for succinate fermentation always contains a variety of nutrient components, the effects of an individual component and the interactions between different components are needed to be studied, which requires rational experiment design. Plackett-Burman design (PBD) and central composite design (CCD) could be used for rapid screening of multifactors to find the most significant factors. Moreover, response surface methodology (RSM) was employed to optimize the concentration of the important factors. Succinate production of *C. glutamicum* in the optimized medium was about 1.46-fold higher than the original medium [59].

Other fermentation parameters such as CO_2_ (or carbonate) supply, temperature, pH, and dissolved oxygen (DO) also showed significant effect on succinate production. As one mole of CO_2_ is theoretically required for the synthesis of one mole of succinate, optimization of CO_2_ partial pressures would contribute to succinate production. Under the optimized conditions, a succinate concentration of 51.6 g/L with a yield of 75.8% was reached for the producer *A. succinogenes* [60]. The culture temperature should be optimized according to the strain employed. As the accumulation of succinate would make the culture broth to be acidic, the pH of the fermentation broth should be adjusted to a suitable value according to maximizing the production. A pH of 6.4 yielded the highest specific succinate productivity for *E. coli* strain AFP111 [61]. The DO control in the whole fermentation process also strongly affected cell growth and succinate production, especially for aerobic cultivation. It was found that 2–5 h of low dissolved oxygen culture during the aerobic phase improved succinate productivity using the metabolically engineered *E. coli* strain SBS550MG [62].

### 6.2. Employment of Different Fermentation Strategies

Most of the current succinate-producing systems are performed under anaerobic conditions. Unfortunately, anaerobic fermentation has inherent disadvantages, for example, long doubling time, slow carbon throughput, and low product formation rates, that are difficult to surmount [63]. Therefore, a “dual-phase” fermentation process was established to overcome these problems. Cells grew quickly to generate enough biomass under aerobic conditions, then switching to anaerobic conditions for succinate production. The volumetric productivity of succinate was greatly enhanced for both *E. coli* NZN111 and AFP111 using the dual-phase culture strategy [64].

Batch or fed-batch fermentation is the preferred mode of operation for succinate production. However, continuous production of succinate is likely to outperform batch processing, especially when considering the projections of the following downstream processing. During the past few years, continuous succinate fermentation has been carried out by researchers from different institutes, but only the natural producers such as *M. succiniciproducens* [65], *A. succiniciproducens* [66], and *A. succinogenes* [67] have been tested. Cell recycle bioreactors with integrated membrane separation system are required for continuous succinate production.

### 6.3. Extension of the Fermentation Feedstocks

Glucose is generally used as the carbon source for succinate fermentation. Even though glucose is abundantly available, its relative high price increases the production costs. In addition, glucose is manufactured by hydrolyzing starch using *α*-amylase and debranching enzyme or isoamylase. The excessive consumption of glucose is a threat to global food security. In order to develop a more sustainable biobased industrial production of succinate, it will be crucial to use low-cost substrates, especially nonfood feedstocks, and develop a robust strain capable of catalyzing these raw materials to produce succinate in good yields.

Glycerol is an abundant waste product of the biodiesel industry with limited commercial uses. The biological production of succinate from glycerol is an attractive process, since it produces a high added-value compound from this by-product while decreasing environmental pollution. Efficient bioconversion of glycerol to succinate has been successfully achieved by using *A. succiniciproducens* [21], *A. succinogenes* [68], and metabolically engineered *E. coli* [69].

Xylose is a renewable sugar which can be derived from lignocellulosic biomass. Fermentation of xylose to succinate has been made possible by current available strains. Anaerobic fermentation of xylose using an engineered *E. coli* strain produced a final succinate concentration of 11.13 g/L [70]. However, using pure xylose for fermentation is obviously economically unfeasible. To further lower the production cost, utilization of biomass hydrolysates, the second-generation fermentation feedstocks, seems to be more significant. There has been an increasing trend towards the utilization of agricultural wastes or residues such as corn straw [71], wheat straw [72], corn stover [73], and sugarcane bagasse [74] for the production of succinate. As most of the succinate-producing strains could not ferment cellulosic biomass, a pretreatment step is required for the conversion of lignocellulosic materials to reducing sugars. The saccharification technology is essential to downstream fermentation [75].

## 7. Separation and Purification of Succinate from the Fermentation Broth

Recovery of succinate from the fermentation broth is the last step for biological succinate production. The separation and purification of succinate are estimated to make up more than 50% of the total costs in its microbial production [76]. To make fermentation-based succinate production competitive with petrochemical processes, the development of optimized producing strains and fermentation processes must be combined with cost-saving and energy-effective downstream processes to minimize the production costs.

**Figure 3:**
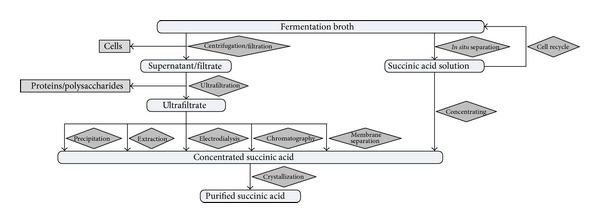
Strategic approaches for the separation and purification of succinate from the fermentation broth.

As shown in Figure 3, the first downstream processing step of succinate purification is to separate microbial cells from the fermentation broth by centrifugation or filtration which is followed by ultrafiltration to eliminate proteins, polysaccharides, and other polymers from the supernatant. For the isolation of purified succinate, different strategies including crystallization, precipitation, extraction, electrodialysis, membrane separation, chromatography, and *in situ* separation have been investigated. The comparison of different downstream processing strategies for succinate separation is presented in Table 3. The traditional succinate recovery method is based on precipitation and crystallization technology. During succinate fermentation, Ca(OH)_2_ can be used to control the fermentation pH and precipitate succinate. In this process, a large amount of CaSO_4_ was accumulated, which is commercially useless [77]. Under acidic conditions, the solubility of succinate is relatively low. Therefore, the classical crystallization method could be used for the separation of succinate [78]. However, the low recovery rate and purity of the final product requires recrystallization to make the final product suitable for commercial use. Among different extraction methods, the reactive extraction of succinate using aliphatic amines as reactive components has been widely reported. The distribution coefficient of succinate in different phases could be easily controlled via adjusting the pH value of the fermentation broth. About 99.8% of purity and 73.1% of recovery rate could be obtained through a newly developed reactive extraction process [79]. However, most of the studies on reactive extraction were performed with pure aqueous phases and the process is often interfered by contaminated acids, impurities, carbon sources, protein, or salts. Electrodialysis is another process capable of separating succinate from nonionized compounds and can be combined with continuous fermentation to realize *in situ* recovery of succinate [80]. The main drawback of this process is its high energy consumption and the expensive and easily polluted membranes. The chromatography methods, especially using ion exchange or adsorption resin, have been recently reported in many studies [81]. An adsorbent resin NERCB09 was found to have a high adsorption capacity of succinate with an excellent recycling property [82]. The same as many other chromatographic processes, the resin used has to be regenerated frequently and the regeneration of sorbent needs a large quantity of acid and alkali, resulting in additional pollutions. As high concentration of succinate would inhibit cell growth, removing the inhibitory product directly from the ongoing fermentation broth might enhance its production. The strategies of extraction, electrodialysis, and chromatographic absorption have the potential to be used for *in situ* separation. *In situ* product removal always requires a specific integrated fermentation system [83]. The requirement of specific bioreactor increases the production costs.

Up to now, separation and purification of succinate from the fermentation broth is still an economical obstacle for its biological production. The downstream processing methods employed by BioAmber for commercial production were mainly based on the classical crystallization strategy [84]. No commercial applications have been reported using the other recovery techniques discussed above since the separation methods studied so far have some drawbacks or limitations. The key challenge to successfully separate succinate from the fermentation broth is how to apply these downstream processing technologies to large-scale industrial processes in a cost- and time-effective manner. In addition, separation process coupled with upstream fermentation, that is, *in situ* product removal, deserves more attentions in the future.

## 8. Conclusions and Future Perspectives

Succinate is finding increasing applications in various areas and biological succinate manufacturing represents a promising path for its viable industrial production. Fermentative succinate production has many advantages, for example, the decreasing consumption of nonrenewable resources and the reduction of greenhouse gas emissions [85]. With the efforts of many researchers in different fields, the current production cost of biobased succinate has been economically competitive with traditional petrochemical processes. However, the extension of biobased succinate as an intermediary feedstock for bulk chemicals production will still rely on inexpensive manufacturing processes. It is estimated that the total production cost of succinate could drop to below $0.45/kg under the perfect condition [86]. The current biological process is still far from this expectation.

The production cost of biobased succinate is largely decided by two factors: the fermentation process and the separation process. As discussed above, downstream processing makes up the majority cost for succinate production. Therefore, downstream processing ranks at the first place to achieve an industrial level production. The development of economically competitive separation technology is essential to reduce the production cost. On the other hand, the quality of the fermentation process will determine the difficulty of separating. The titer of succinate and the content of other organic acids in the fermentation broth strongly affect the separation process. Microbial strains with improved succinate-producing ability and lower by-products formation allow for simpler and less expensive purification methods. Recent advances in synthetic biology and systems biology have provided new tools to construct more efficient succinate-producing strains [87]. In addition, improvements of the fermentation process and its integration with the separation system also have the potential to lower the whole production cost. It must be admitted that advances in biobased succinate production could only be achieved under the cooperation of scientists having different background and expertise.

Obviously, fermentative succinate production opens a new direction for bulk chemicals manufacture. Although a number of technical challenges must be overcome, the development of this biological process has a long term prospect. We believe that by the joint efforts of researchers in different fields, a sustainable and economically attractive biobased succinate production will finally replace the traditional petrochemical processes in the near future.

## Figures and Tables

**Table 1:** The typical microorganisms used for fermentative succinate production.

	Type	Species	Oxygen requirement
Natural producers	Bacteria	*Actinobacillus succinogenes *	Facultative anaerobe
	Bacteria	*Anaerobiospirillum succiniciproducens *	Strict anaerobe
	Bacteria	*Mannheimia succiniciproducens *	Facultative anaerobe
	Bacteria	*Bacteroides fragilis *	Strict anaerobe
	Bacteria	*Enterococcus faecalis *	Facultative anaerobe
	Bacteria	*Klebsiella pneumoniae *	Facultative anaerobe
	Bacteria	*Succinivibrio dextrinosolvens *	Strict anaerobe
	Fungi	*Aspergillus niger *	Facultative anaerobe
	Fungi	*Paecilomyces varioti *	Facultative anaerobe
	Fungi	*Penicillium simplicissimum *	Facultative anaerobe

Engineered producers	Bacteria	*Escherichia coli *	Facultative anaerobe
	Bacteria	*Corynebacterium glutamicum *	Aerobe
	Yeast	*Saccharomyces cerevisiae *	Facultative anaerobe

**Table 2:** Overview of metabolic engineering strategies to improve succinate production using different microorganisms.

Strains	Genotypes	Culture strategies	Succinate production	References
*Escherichia coli *				

SBS110MG	*ΔadhE, ΔldhA, expression of Lactococcus lactis pyc *	Batch	15.6 g/L, 1.3 mol/mol glucose	[32]
NZN111	*ΔpflB, ΔldhA *	Fed-batch	28.2 g/L, 1.13 mol/mol glucose	[33]
AFP111	*ΔldhA, ΔptsG *	Batch	0.88 mol/mol glucose	[34]
W3110GFA	*ΔptsG, ΔpykF, ΔpykA *	Batch	17.35 mM	[35]
QZ1111	*ΔptsG, ΔpoxB, Δpta, ΔsdhA, ΔiclR *	Batch	1.45 mmol/(g·h)	[36]
HL27659k	*ΔsdhAB, ΔackA-pta, ΔpoxB, ΔiclR, ΔptsG *	Fed-batch	0.91 mol/mol glucose	[37]
KJ073	*ΔldhA, ΔadhE, ΔackA, ΔfocA-pflB, ΔmgsA, ΔpoxB *	Batch	1.2 mol/mol glucose	[38]
JCL1208	*overexpression of native ppc *	Batch	10.7 g/L	[39]
xz320	*ΔackA, ΔldhA, ΔadhE, ΔpflB, ΔmgsA, ΔpoxB, Δppc *	Fed-batch	348 mM	[40]
LS1	*ΔldhA, overexpression of native mae *	Batch	2.34 g/L	[41]
NZN111	*ΔpflB, ΔldhA, overexpression of native mdh *	Fed-batch	15.2 g/L	[42]
NZN111	*ΔpflB, ΔldhA, overexpression of native mae and fum *	Batch	7 g/L	[43]
SBS990MG	*ΔadhE, ΔldhA, Δpta-ackA, expression of Lactococcus lactis pyc *	Batch	1.7 mol/mol glucose	[44]
NZN111	*ΔpflB, ΔldhA, overexpression of native pncB *	Fed-batch	18.3 g/L	[45]
K-12*ppc *	*Δppc, expression of Actinobacillus succinogenes pck *	Batch	26.4 mM	[46]

*Saccharomyces cerevisiae *				

AH22ura3	*Δsdh1, Δsdh2, Δidh1, Δidp1 *	Batch	3.62 g/L, 0.11 mol/mol glucose	[16]
DFRDS	*ΔOSM1, ΔFRDS *	Batch	130 *μ*mol/(h·g dry cells)	[47]
8D	*Δsdh, Δser3/ser33, Overexpression of native icl1 *	Batch	0.9 g/L	[48]

*Corynebacterium glutamicum *				

BL-1	*Δsdh, Δpta-ackA, Δpqo, Δcat, overexpression of native ace, pyc and ppc *	Fed-batch	10.6 g/L, 0.45 mol/mol glucose	[17]
BOL-2	*Δcat, Δpqo, Δpta-ackA, ΔldhA, overexpression of native pyc and Mycobacterium vaccae fdh *	Fed-batch	1134 mM, 1.67 mol/mol glucose	[49]

*Mannheimia succiniciproducens *				

LPK7	*ΔldhA, ΔpflB, Δpta, ΔackA *	Fed-batch	52.4 g/L, 1.16 mol/mol glucose	[50]

**Table 3:** Comparison of different downstream processing strategies for succinate separation and purification.

Downstream processing strategies	Advantages	Disadvantages
Crystallization	Easy to be operated; without additional reagents.	Low succinate yield and purity; recrystallization is required.
Precipitation	Low technological barriers; inexpensive precipitants.	Requirement of large quantities of precipitants; generation of useless by-products.
Extraction	High output and low energy consumption.	Pretreatment of the fermentation broth is required; expensive extraction agents used for reactive extraction.
Electrodialysis	Relatively mild conditions; can be used for continuous separation.	High energy consumption; high cost of the membranes; low selectivity for succinate.
Chromatography	Easy to be scaled up.	Regeneration of the chromatographic matrix requires large amounts of acids and alkalis.
*In situ* separation	Can be coupled with the fermentation process; relieving product inhibition.	Relatively complicated processes; regeneration of separation sorbent is required.
